# Depression and playfulness in fathers and young infants: A matched design comparison study

**DOI:** 10.1016/j.jad.2017.12.107

**Published:** 2018-03-15

**Authors:** Vaheshta Sethna, Lynne Murray, Olivia Edmondson, Jane Iles, Paul G. Ramchandani

**Affiliations:** a Department of Forensic and Neurodevelopmental Sciences, Institute of Psychiatry, Psychology and Neuroscience, King's College London, London, UK; b School of Psychology and Clinical Language Sciences, University of Reading, Reading, UK; c Stellenbosch University, Stellenbosch, South Africa; d Academic Unit of Child and Adolescent Psychiatry, The Centre for Mental Health, Imperial College London, London, UK

## Abstract

**Background:**

Depression in fathers in the postnatal period is associated with an increased risk of some adverse child developmental outcomes. One possible mechanism for the familial transmission of risk is through the negative effects of depression on parenting and the parent-child relationship. So far, evidence indicates that depressed fathers tend to be more withdrawn in their early interactions. However, the interaction dimensions studied to date may not be able to detect and accurately classify unique features of father-infant play – including physically stimulating and highly rousing episodes of play. Hence, in this matched design comparison study, we set out to examine, for the first time, links between diagnosed paternal depression in the postnatal period and playfulness in father-infant interactions.

**Methods:**

Fathers and their infants were assessed when the infants were 3 months old. Paternal depression was diagnosed using a structured psychiatric interview. Currently depressed (n = 19) and non-depressed (n = 19) fathers were individually matched on age and education. Fathers were filmed playing with their children. Four dimensions were coded for paternal playfulness during free-play: physicality, playful excitation, tactile stimulation and active engagement.

**Results:**

Depressed fathers, compared to non-depressed fathers, engaged in fewer episodes of playful excitation (mean scores: 0.71 vs.2.53, *p* = 0.005), less gentle touch (mean time: 38.57 vs. 53.37, *p* = 0.015) and less active engagement (mean scores: 2.29 vs 3.24, *p* = 0.044). When controlling for infant fretfulness, the findings remained largely unchanged.

**Limitations:**

The sample size was small and the sample was limited to mostly white, well-educated fathers.

**Conclusions:**

Playful paternal behaviours as early as 3 months differ between fathers with and without depression. These changes may help in understanding children’s risk in relation to paternal psychopathology and could be a target for future family interventions.

## Highlights

• Playful behaviours at 3-months differ between depressed and non-depressed fathers.
• Depressed fathers use less playful excitation, gentle touch and active engagement.
• Coding measures focusing on fathers may accurately detect and classify playfulness.
• Unique features of paternal play could be a target for future family interventions.

## 1 Introduction

### 1.1 Paternal depression and father-infant interactions

A developing body of literature now documents that mood disorders are not limited to mothers in the postnatal period. Fathers experience depressive symptoms as well, with prevalence rates of around 5–10% within the first year of a child's life (for reviews see, Cameron et al., 2016; Edward et al., 2014; Paulson and Bazemore, 2010). Furthermore, depression in fathers has been linked with multiple indicators of adverse child outcome, including behavioural and emotional problems, impaired cognitive development, and an increased risk of the development of psychopathology (Barker et al., 2017). One possible mechanism for the familial transmission of risk is through its negative effects on parenting and the father-child relationship (Kane and Garber, 2009, Nath et al., 2016, Sethna et al., 2015, Sethna et al., 2012).

So far, evidence indicates that paternal depression is associated with less optimal father-infant interactions with a few exceptions (Field et al., 1999, Lundy, 2002). For example, in comparison to non-depressed fathers, depressed fathers are less *responsive* in free-play with their 3-month old infants (Parfitt et al., 2013), tend to engage in fewer *activities*, such as, outdoor play, singing or reading to their children aged 9-months (Paulson et al., 2006), *touch* their 4-month old infants less frequently during routine tasks (Zaslow et al., 1985), and are less *engaged* during free play at 3 months and 12 months (McElwain and Volling, 1999, Sethna et al., 2015).

In general, while these studies have linked depressive symptoms to more withdrawn paternal behaviours, they also highlight an important limitation. That is, the scales employed to code father-infant interactions have usually been developed using mother–infant dyads. Hence, while they may be particularly sensitive to the interaction style of mothers; they may not be able to detect and accurately classify unique features of father-infant interactions (McElwain and Volling, 1999, Paquette and Dumont, 2013, Sethna et al., 2015). For instance, episodes of sudden vocal and behavioural activity in close proximity to the infant are coded as *‘intrusive’* using a maternal coding scheme (McElwain and Volling, 1999, Sethna et al., 2015). However, in the context of father-infant interactions, these playful behaviours (which excite, surprise, and stimulate children) tend to promote attention and positive communication between the dyad. Consequently our understanding of early parenting by fathers is incomplete due to the conventional (though changing) maternal focus on parenting behaviours.

We have previously reported that fathers with depression are less intrusive, or more withdrawn, in their interactions (Sethna et al., 2015). Like much previous research, a measure of parent-child interaction that had been developed for mothers was used. Hence we were restricted to studying the key interaction dimensions typically examined in relation to mother- infant interactions. In this study, we extend previous work with depressed fathers by using a measure of parent-child interaction mainly developed to code paternal playful behaviours. Since playfulness comprises a greater proportion of fathers’ interaction with children than it does for mothers (Craig, 2006). Thus, the current study examines, for the first time, links between diagnosed paternal depression in the postnatal period and playfulness in father-infant interactions with 3 month old infants.

### 1.2 What play style do fathers engage in?

Early research on father–infant relations focused primarily on the interaction styles of fathers compared to mothers, and conclusions were made in the 70's that fathers were not simply a replacement for mothers - their interaction styles were qualitatively different (Belsky, 1979, Clarke-Stewart, 1978, Lamb, 1977, Weinraub and Frankel, 1977, Yogman, 1977). In subsequent research, father-infant interactions were described as less predictable and highly rousing episodes of behaviours - comprising a rapid increase and decline of emotional excitement. In contrast, maternal interactions comprised regulated sequences of social exchange including gaze, vocalizations and affect. Moreover, while mothers were more likely to follow their infant's focus of attention, fathers, in contrast, appeared more directive and disruptive (Belsky et al., 1984, Power, 1985, Stevenson et al., 1988, Yarrow et al., 1984).

More recent reports suggest that mothers and fathers adopt multiple roles in the family system (Tamis-LeMonda, 2004). For instance, both parents may function as caregivers, role-models, teachers, breadwinners and spouses - determined by, for instance, work arrangements, family structure, parent's preferences, obligations and health needs. As a result of these multiple roles, similarities may exist in the way that both parents interact with their children (Parfitt et al., 2013, Roggman et al., 2004). For example, fathers who are the primary care-givers, tend to be more comforting, sensitive and engage in less physical play (Lamb et al., 1982, Lewis et al., 2009). In their complimentary influences, fathers are likely to perform roles played by mothers, and vice versa, in response to family circumstances that entail adjustment (Cabrera et al., 2014). Nonetheless, differences in the way mothers and fathers interact with their children are also evident - such that, on average, fathers tend to engage in more physical and competitive play routines (Stgeorge and Freeman, 2017). Notably, fathers engage more frequently than mothers in vigorous physical play from the first year of their child's life (MacDonald and Park, 1986). This increase in the prevalence of more active play in fathers has been linked to socio-emotional development (Paquette and Dumont, 2013), and recent meta-analytic evidence points towards positive links with multiple domains of children's behaviour, including, social competence, emotional development and the ability to self-regulate (Stgeorge and Freeman, 2017).

Hence, in this investigation, we focus on playfulness that characterises father-infant interactions. Based on existing evidence, playfulness is operationalized and measured by the following four interaction domains: (i) physicality (gross motor stimulation) (ii) playful excitation (sudden, unexpected verbal or non-verbal behaviours), (iii) tactile stimulation (touch), (iv) active engagement (stimulation via paternal behaviour, affect, facial expression, and tone of voice).

### 1.3 The impact of depression on dimensions of playfulness in father-infant interactions

Play^1^* is a significant component of the parent-infant relationship and an important context for infant learning (Teti et al., 1988). Physicality in play during the first six months, corresponds to neuromuscular maturation, evident in the improved control of specific motor patterns, and in the subsequent development of socialization skills (Pellegrini and Smith, 1998). Father-infant interactions can also encompass episodes of ambiguous, unexpected behaviours (e.g., unpredictable movements within the infant's visual field or a change in pitch and volume of voice) (Dixon et al., 1981, Labrell, 1994). Although not empirically tested in infancy, there are suggestions of a positive association between these rousing play episodes and the decoding of emotion (happiness, sadness, anger, fright and neutrality) in pre-school children (Carson et al., 1993).

We are not aware of specific evidence of the impact of parental depression on physicality in play and sudden excitatory arousal or playfulness. It is, however, well recognized that depression impacts on behaviours including linguistic production (rate, volume, and pitch), facial expression, gaze, posture and gestures (Segrin, 2000). In view of these findings and those which suggest that depressed fathers tend to be more withdrawn in their early interactions, we predict that depressed fathers will be less physical **(Hypothesis 1)** and engage in fewer episodes of playful excitation **(Hypothesis 2)** when interacting with their infants, compared to non-depressed fathers.

Touch serves a communicative function, signalling emotions of positive (Pelaez-Nogueras et al., 1996) and negative valence (Hertenstein and Campos, 2001), and in the transmission of more specific messages, for example, security in the presence of the caregiver (Tronick, 1985). Research on tactile stimulation in father-infant interactions is very limited (Shields and Sparling, 1993, Zaslow et al., 1985). For example, Zaslow and colleagues (1985) have shown that in the context of paternal ‘blues’ fathers show diminished contact with their infants aged 4 months. While studies from the maternal literature similarly report that depressed compared to non-depressed mothers spend less time touching their infants, they also report that depressed mothers use more *negative* touch (for e.g. poking, shaking, tickling, pulling or tugging limbs) as opposed to *positive* touch (gentle stroking, or playfulness) (Ferber, 2004, Herrera et al., 2004, Malphurs et al., 1996b). We aim to investigate differences in the quantity and quality of tactile stimulation, i.e*. touch* in depressed and non-depressed fathers. Based on the available evidence, we predict that depressed fathers in comparison to non-depressed fathers will spend less time touching their infant, and are more likely to engage in negative (i.e. vigorous) touch, as opposed to positive (i.e. gentle) touch **(Hypothesis 3).**

Active stimulation using a range of modalities during communication helps modulate infant arousal and reinforces behaviours. Within this cycle, an infant responds to parental stimulation with increased attention, communication and positive affect (Bridges and Connell., 1991, Bridges et al., 1997, Diener et al., 2002, Yarrow et al., 1984). For example, Bridges et al. (1997) have found that, in the presence of an actively communicating father, infants displayed positive affect and were generally more communicative. Infant developmental outcomes studied in relation to an early animated environment include, for example, emotional regulation, cognitive competence, and exploration and motivation (Clarke-Stewart, 1978, Diener et al., 2002, Yarrow et al., 1984). So far, the impact of depression on paternal use of animation/stimulation has not been specifically addressed. We predict that depressed mood will interfere with the father's ability to stimulate the infant vocally, physically, emotionally and psychologically- their interactions will be quiet and contained **(Hypothesis 4)**.

## 2 Methods

### 2.1 Study design

This cross-sectional matched sample study comprised a total of 38 fathers. Matching variables included age and education in line with previous research (Tamis-LeMonda et al., 2004). We had 19 fathers with current major depressive disorder in the study, and then identified and individually matched fathers without current depressive disorder and with equivalent or the most similar values of the two covariates. This created a new sample of matched participants − 19 fathers with current major depressive disorder and 19 fathers without depression - previously cited (Sethna et al., 2012).

The matching procedure was based on a random order nearest available matching method (Rosenbaum and Rubin, 1985, Rubin, 1979). The first depressed participant was randomly matched with the closest non-depressed participant (N), followed by the second depressed participant being matched with the yet unmatched closet non-depressed participant (N-1), and so on, until all depressed fathers were matched. By construction, the currently depressed and non-depressed fathers in the matched sample had identical (or nearly identical) values of age and education.

### 2.2 Setting

Participants were drawn from a larger prospective cohort study examining the impact of paternal depression on child development (Ethical approval: 06/Q1604/63). They were recruited from postnatal wards in Oxfordshire and Buckinghamshire UK, and assessed at 3, 12 and 24 months. For this study, exposure and outcome data were concurrently assessed at the 3-month time point.

### 2.3 Participants

All fathers recruited into the study met inclusion criteria of being 18 years or older, speaking English, being married or in a stable relationship and residing with their infant. Infants were of gestational age >36 weeks, birth weight ≥ 2 kg, and had no major congenital anomalies.

Seven weeks after the birth of their child all consenting fathers were sent a questionnaire including the Edinburgh Postnatal Depression Scale (EPDS, Cox et al., 1987). All the fathers screening positive (10 or above on the EPDS), and a random one-in-four sample of those screening negative (an EPDS score less than 10) were subsequently invited to take part in the study. Those agreeing to participate further, were visited at home when their infant was approximately 14 weeks old. During this visit fathers were interviewed using the Structured Clinical Interview for DSM-IV (SCID; First et al., 2002) to establish whether they met criteria for major depressive disorder - as previously reported elsewhere (Edmondson et al., 2010, Ramchandani et al., 2011).

Subsequently two study groups were identified: (i) currently depressed – including fathers diagnosed as experiencing current major depressive disorder using the SCID, (ii) non-depressed – including fathers with neither current nor past diagnosis of major depressive disorder on the SCID or elevated symptoms on the EPDS screening questionnaire.

### 2.4 Variables

Exposure: Diagnosed paternal depression

Outcome: Four categories of behaviour that characterize father-infant interaction including: (i) Physicality in play, (ii) Playful excitation, (iii) Tactile stimulation and (iv) Active engagement.

### 2.5 Data source and measurement

Data for this study were collected three months post birth. All assessments were conducted in the families’ homes during times when the infants were typically alert and fed. Father – infant dyads were videotaped during two interactive settings, including 3 min of free play, which was the focus of this investigation. Fathers were asked to place their infants in a supine position on a floor-mat, and to play with and talk to their infant, as they would normally do, without the use of toys. Parents were asked not to allow their children to use pacifiers during the videotaping so that the researchers could hear children's vocalizations. Following the videoed observational assessment, all fathers were interviewed using a structured psychiatric interview. The main study variables were measured using the following tools:

#### 2.5.1 Paternal depression (exposure variable)

The Structured Clinical Interview for DSM-IV Axis 1 Disorders (SCID; First et al., 2002), was used for diagnosing major depressive disorder. The SCID has been found to have high reliability (Zanarini et al., 2000) and validity (Basco et al., 2000). All interviews were conducted by trained graduate psychologists or psychiatrists.

#### 2.5.2 Father-infant interactions (outcome variables)

Four categories of behaviour that characterize father-infant interaction were coded and detailed below (manual available from the authors upon request). They include: (i) Physicality in play, (ii) Playful excitation, (iii) Tactile stimulation, (iv) Active engagement.

##### 2.5.2.1 Physicality in play

Gross-motor stimulation (movement of infant limbs and body position), coded using a 5 point Likert scale of measurement. The scale comprises progressive levels of physicality from gross motor energetic stimulation, whereby the infant is lifted off the mat (5) to low-energy physical stimulation, holding of the infant's arm/legs with minimal movement (1). Thus coding is based on progressive levels of physicality, with 5 as the highest level of physicality in play. This scale is modified from previous work on fathers by Crawley and Sherrod (1984) with 10–13 month old infants, and by Yogman (1981) with infants in the first six months of life.

##### 2.5.2.2 Playful excitation

A frequency count of sudden, emotional, unpredicted and repetitive short sequences / and or single occurrence of behaviour. This was adapted from previous work in which paternal use of teasing entails ‘unexpected ambiguous behaviours which destabilize the infant and whose expectations are contradicted’ (Labrell, 1994, p. 128). In this category of playful behaviour we code for specific instances of unpredictable movements within the infant's visual field, change in pitch and volume of voice quality which stimulate and arouse the infant. Examples of arousal behaviours coded include: sudden paternal vocalization, i.e. high pitched speech or non-verbal exclamation, unpredicted physical activity around the infant or distal stimulation by motor movements and high positive arousal through sudden animated facial or vocal expression. Higher scores suggest the increased episodes of arousal behaviours and hence increased playful excitation during interactions.

##### 2.5.2.3 Tactile stimulation

The quantity of overall touch (i.e. total time in seconds spent in physical contact with the infant during the 3 min interaction), and the quality of touch (i.e. the proportion of time, measured in seconds, spent in a certain type of touch) were both coded.

Quantity of overall touch comprised a rating of the proportion of the interaction (i.e. the total time in seconds across the entire play episode) during which the father touches the infant either as part of a game; to gain or maintain the infant's attention, as an affectionate gesture or in response to the infant's physical movements. In line with previous studies we used proportions to account for any differences in the overall duration of the interaction. Higher scores indicate increased time spent in tactile behaviours.

Quality of touch was based on previous work (Moreno et al., 2006, Polan and Ward, 1994). The mean time spent in each of the following touch items was measured: (i) Inactive touch (ii) Light touch (iii) Firm touch (iv) Stimulating touch and (v) Active touch and (vi) Practical touch.

Individual touch items were also combined into two composites, and subsequently used for the current analyses: *Gentle touch -*the mean time (in seconds) spent in inactive touch, light touch and practical touch. This variable corresponds to previous research (Feldman and Eidelman, 2003, Ferber et al., 2008, Pelaez-Nogueras et al., 1996). V*igorous touch* -the mean time (in seconds) spent in firm, stimulating and active touch, also comparable to previous studies (Stack and Muir, 1992, Weiss, 1988, Weiss and Wilson, 2006).

##### 2.5.2.4 Active engagement

The level of vitality and liveliness within the session, coded through paternal behaviour, affect, facial expression and tone of voice. This dimension taps into vocal, emotional, psychological and physical availability of the parent- the parent effectively communicates his awareness of the infant's presence, is receptive to ongoing communication and is available to respond or initiate social interaction empathically and appropriately. Rated on a likert scale of measurement from 1 to 5; higher ratings (5) indicate increased stimulation throughout the play session with momentary episodes of calm to regulate arousal, and lower scores (1) indicate minimal use of stimulation. Active engagement is a reflection of the effort the father puts into the interaction to create a lively, vigorous environment (higher scores) as opposed to a quiet and contained one (lower scores).

All videotapes were coded by two researchers blind to paternal depression status. Inter-rater reliability between the two coders was established using Intra-class coefficients (ICC) on interval data (Playful excitation: ICC = .97; Touch types: ICC ranged from .74 to .92), and weighted Kappa on ordinal data (active engagement: k = 0.78; physicality in play: k = 0.81).

#### 2.5.3 Infant fretfulness (covariate)

Observational measures of infant behaviour were coded using The Global Rating Scales for Mother– Infant Interaction (GRS, Murray et al., 1996). The GRS comprise individual infant behaviours rated on five point scales. We assessed ‘Infant fretfulness’, which comprised two infant behaviour scales from the GRS: (i) happy–distressed and (ii) non-fretful–fretful.

### 2.6 Bias

Sampling bias in the current study was minimized as the non-depressed fathers were selected from the same community sample which included the depressed fathers. They differed mainly on the exposure under consideration. Recall bias was minimized as participants completed the EPDS screen 7 weeks after the birth of their child.

### 2.7 Study size

The sample size for the current study comprised all the depressed fathers (from the wider prospective cohort study) with diagnosed depression on the SCID (n = 19) who were then individually matched with a non-depressed father.

### 2.8 Statistical methods

First, the sample is described – demographic characteristics of the total sample were examined using means and standard deviations (SD) for continuous data and percentages for categorical data. We also examined infant (gender and birth order) and paternal demographic characteristics (age, marital status, educational attainment and ethnicity) in relation to the two study groups. Second, descriptive statistics for the interaction dimensions are presented and bivariate associations between these dimensions were tested. Third, using the Wilcoxon-signed-rank test for paired data, depressed and non-depressed fathers were compared on the four main interaction dimensions. This non-parametric test was used as the sample was small and some data were not normally distributed. Finally, where a significant association was found between exposure (paternal depression status) and outcome variables (four interaction domains of paternal playfulness), we additionally took into consideration the infant's affective state, i.e. fretfulness. This was based on existing evidence which suggests that parental depression is linked with difficult child temperament (Hanington et al., 2010), and that negative child affect is also linked with less positive parent- child interactions (McBride et al., 2002). We therefore wished to try and control for any potential effects of having a more difficult or fretful infant on the interaction, so that we could more confidently ascribe any differences in interaction to the potential effects of depression in the father. Our rationale for including infant fretfulness was thus based on the existing literature.

## 3 Results

### 3.1 Depression and infant and paternal demographic characteristics

Fathers (n = 38) had a mean age of 35.89 years (*SD* = 5.42), 94.7% were white and all were married/cohabiting. In terms of highest level of educational attainment, 10.5% had GCSE's, and 21.1% had post-graduate qualifications; 92.1% of the fathers were in full time employment. For all but two fathers, English was their first language. Of the infants, 18 (47.4%) were first born and 14 (36.8%) second born.

There were no differences between the two groups on infant (gender and birth order) and paternal demographic characteristics (marital status, ethnicity and employment status) (Table 1). Additionally, infant fretfulness did not differ between depressed (*M* = 3.74, *SD* = 1.32) and non-depressed fathers (*M* = 3.94, *SD* = 1.09); *t* = −0.50, *p* = 0.619.

**Table 1:** Comparison of paternal and infant demographic characteristics by paternal diagnostic status.

**Sample characteristic**	**Currently depressed (n = 19)**	**Non-depressed (n = 19)**	**Statistic**
**Father's age** (years)	36.26 (5.94)	34.11(8.93)	t (36) = .877, *p* = 0.387
**Father's education level**			
No qualification (%)	5 (26.3%)	6 (31.6%)	χ2 = .334, *p* = 0.841
Diploma (%)	4 (21.1%)	2 (10.5%)	
College Degree (%)	6 (31.6%)	7 (36.8%)	
Postgraduate (%)	4 (2.1%)	4 (21.1%)	
**Marital status**			
Single (%)	–	–	–
Married (%)	19 (100)	19 (100)	
**Ethnicity**			
White (%)	18 (94.7%)	17 (94.4)	χ2 = .002, *p* = 0.969
Non-white (%)	1 (5.3%)	1 (5.6)	
**Infant Gender**			
Female (%)	12 (63.2%)	12 (63.2%)	χ2 = .00, *p* = 1.00
Male (%)	7 (36.8%)	7 (36.8%)	
**Birth Order**			
First	8 (42.1%)	9 (47.4%)	χ2 = 3.13, *p* = 0.373
Second	8 (42.1%)	7 (36.8%)	
Third	1 (5.3%)	3 (15.8%)	
Fourth	2 (10.5%)	0 (0%)	
**Infant fretfulness**	3.74 (1.32)	3.94 (1.09)	t (36) = −0.50, *p* = 0.619

### 3.2 Correlations between the four behavioural categories

Descriptive statistics for the interaction dimensions and bivariate associations between them are presented in Table 2. Fathers who used more gross motor stimulation in their play tended to touch their infant vigorously (*r*_*s*_ = 0.35, *p* < 0.05). Furthermore, fathers who engaged in increased playful excitation touched their infant less vigorously (*r*_*s*_ = −0.37, *p* < 0.05), and their interactions were highly stimulating (*r*_*s*_ = 0.53, *p* < 0.01).

**Table 2:** Descriptive statistics and intercorrelations among the dimensions of play (N = 38).

	***M***	***SD***	***Mdn***	***Range***	***Min.***	***Max.***	1	2	3	4	5
1. Physicality in play^a^	3.52	1.34	4	4	1	5	1.00				
2. Playful excitation^b^	1.67	1.92	1	6	0	6	0.03	1.00			
3. Gentle touch^c^*(composite)*	46.38	17.03	47.21	77.33	6	83.33	−0.32	0.30	1.00		
4. Vigorous touch^c^*(composite)*	22.38	15.14	25.00	84.45	0.55	85.00	0.35*	−0.37*	−0.34*	1.00	
5. Active engagement^a^	2.80	1.33	3	4	1	5	0.31	0.53**	−.12	.07	1.00

* p<.05, **p<.01. *M* = Mean, *SD* = Standard Deviation, *Mdn* = Median.

a Likert scale of 1-5.

b Frequency count of occurrence.

c Time (seconds) spent in the specified touch type.

### 3.3 Differences in the interactions of depressed and non-depressed fathers

#### 3.3.1 Physicality in play and playful excitation

Depressed and non-depressed fathers did not differ in their use of physicality during play (Table 3). Findings on episodes of playful excitation indicate that depressed fathers were less likely to arouse and startle their infants using sudden, emotional, physical or vocal behaviours (*M* = 0 .71 = SD = 1.16) compared to non-depressed fathers (*M* = 2.53, *SD* = 2.03); *z* = −.280, *p* = .005, with a medium effect size (*r* = 0.45).

**Table 3:** Interaction categories, by paternal diagnostic group.

	Non-depressed (n = 19)	Depressed (n = 19)	*Z*^a^	*p*
Physicality in play^c^	4.00 (4.00)	4.00 (3.00)	−.16	.874
Playful excitation^b^	2.00 (6.00)	0.01 (3.00)	−2.80	.005
Active engagement^c^	4.00 (4.00)	2.00 (4.00)	−2.01	.044

Data are given as median (range).

a Wilcoxon signed ranks.

b Frequency count of occurrence.

c Likert scale of 1–5 (higher ratings indicate increased episodes of behaviour).

#### 3.3.2 Tactile stimulation

There were no differences in the overall duration of touch in the two study groups (Table 4). However, depressed fathers were less likely to engage in *gentle touch* (*M* = 38.57, SD = 16.11) compared to non-depressed fathers (*M* = 53.37, SD = 14.97); *z* = −2.44, *p* = .015, with a medium effect size (*r* = 0.40). There were no mean differences in the vigorous touch composite between the two study groups (Table 4).

**Table 4:** Tactile stimulation, by paternal diagnostic group.

	Non-depressed (n = 19)	Depressed (n = 19)	*Z*^a^	*p*
Quantity of touch^b^	79.76 (45.92)	76.64 (62.22)	−.36	.723
Quality of touch				
1. Gentle touch composite^c^	50.33 (50.56)	44.00 (52.88)	−2.44	.015
2. Vigorous touch composite^d^	19.99 (48.45)	31.66 (83.34)	−1.54	.124

Data are given as median (range).

a Wilcoxon signed ranks.

b Percentage of the total time, i.e. three minutes.

c Time spent in inactive touch, light touch and practical touch.

d Time spent in firm, stimulating and active touch.

#### 3.3.3 Active engagement

As shown in Table 3, depressed fathers (*M* = 2.29, *SD* = 1.31) compared to non-depressed fathers (*M* = 3.24, SD = 1.15) were less actively engaged with their infants; *z* = −2.01, *p* = .044, with a medium effect size (*r* = 0.33).

### 3.4 Controlling for infant fretfulness during interactions

Next, when adjusting for infant fretfulness, group differences reported on the following dimensions remained largely unchanged: playful excitation (unadjusted: *β* = 0.48, *p* = 0.004; adjusted: *β* = 0.48, *p* = 0.004); gentle touch (unadjusted: *β* = 0.46, *p* = 0.006; adjusted: *β* = 0.46, *p* = 0.007); active engagement (unadjusted: *β* = 0.34, *p* = 0.044; adjusted: *β* = 0.36, *p* = 0.038).

## 4 Discussion

This study aimed to broaden the focus of measurement of early paternal play behaviours in the context of depression. The findings afford new, albeit exploratory, insights into the impact of depression on: (i) physicality in play, (ii) playful excitation, (iii), tactile stimulation, (iv) active engagement in fathers. The findings suggest that depressed fathers compared to non-depressed fathers engaged in fewer episodes of playful excitation, less gentle touch and less active engagement. There was no evidence of a difference in physical play and in the overall duration of tactile engagement between the two study groups.

The study does have several limitations, including a small sample size, and limited demographic variability of the participants (predominantly white, well-educated fathers). Furthermore, we did not conduct a formal power analysis in advance of the study. It was anticipated that the exploratory nature of the study would subsequently allow for reported group differences from the small sample, with clearly defined groups, to be then tested on a larger sample. Hence caution should be exercised in interpretation of the findings reported, at least until subsequent research also addresses these important questions.

In addition there are a number of strengths to the current study. First, we developed these specific dimensions of paternal behaviour drawing on the existing literature on child developmental and parenting strategies, to develop relevant constructs. Second, the scales were reliably administered and coded by blind coders. Third, the study includes two well-matched groups, and participants were diagnosed for depression using a structured interview rather than a questionnaire for symptoms. Although, the purpose of matching was to eliminate bias in relation to paternal age and education, future studies, with larger samples should consider additional covariates. There are many potential covariates to consider, but some key ones might include infant birth order, birth weight, child gender and maternal depression (Hallers-Haalboom et al., 2017, Vismara et al., 2016).

The prediction that depressed fathers would engage in less physical play compared to non-depressed fathers was not supported. These findings stand in contrast to previous studies of fathers and their infants in play across the first year of life (Clarke-Stewart, 1978, , 1984, Lamb, 1977, Yogman, 1981). We suspect that, at three months, face to face communication in fathers may focus on tactile behaviours and limb movement games rather than active gross-motor stimulation. Previous research suggests that only in the sixth month after birth is there a peak in gross-motor stimulation (Pellegrini and Smith, 1998), while other studies seem to suggest low levels of physical play before 1 year (MacDonald and Park, 1986). Furthermore, fathers tend to engage in physical play with their infant sons more than their daughters (Bronstein, 1984). Hence, the lack of group difference reported on physicality in play, may be due to our sample comprising more female infants overall. Moreover, our study may lack the power to further differentiate boys and girls, and hence child gender would be important to consider in future studies with larger samples.

Although positive arousal in paternal interactions has been consistently reported (Arco, 1983, Clarke-Stewart, 1978, Feldman, 2003, Yogman, 1981), we are not aware of previous evidence on the influence of paternal depression on excitatory features of father-infant interactions. The prediction that depressed fathers compared to non-depressed fathers would engage in fewer episodes of playful excitation was supported in the present study. It is likely that the display of sudden vocal, motoric and facial expressions in the context of high emotionality may be more susceptible to depressive affect. Further, that such behaviours are more likely to be produced as a result of the fathers’ own level of engagement and interest in the interaction, and not in response to a prior behaviour emitted by the infant, suggests they may represent a distinct motivational characteristic of social communication, which is reduced in fathers experiencing depression.

In contrast to findings from the maternal literature (Herrera et al., 2004, Malphurs et al., 1996a), we did not find a difference in the overall duration of touch between depressed and non-depressed fathers. Earlier findings from this study suggest that depressed and non-depressed fathers did not differ in sensitivity in their interactions (Sethna et al., 2015). These findings may thus help explain why depressed and non-depressed fathers did not differ on the duration of tactile stimulation. Furthermore, in early infancy, touch facilitates the regulation of arousal, attentiveness and interest, and helps soothe the infant (Koester et al., 1989, Tronick and Cohn, 1989). Since infants in the two study groups were similar with respect to positive affect and attentiveness, it is likely that fathers did not differ in the overall duration of tactile stimulation necessary to regulate infant behaviour.

The prediction that depressed fathers compared to non-depressed fathers would engage in more vigorous touch as opposed to gentle touch was partially supported. We found that while there were no differences in the use of vigorous touch by depressed and non-depressed fathers, they did differ in their use of gentle touch. These results are partially consistent with previous findings from the maternal literature (Cohn et al., 1986, Feldman, 2003, Field et al., 1990, Weinberg and Tronick, 1998), which indicate an association between depression and more negative tactile behaviours as opposed to affectionate ones.

Finally, depressed fathers compared to non-depressed fathers engaged in fewer episodes of active engagement. The few available studies on the impact of depression on paternal behaviour report similar findings with regards to paternal withdrawal and lack of engagement (McElwain and Volling, 1999, Paulson et al., 2006, Zaslow et al., 1985). Depression is likely to interfere with the parent's emotional availability to stimulate the infant, evident in laboratory studies that report fewer positive animated faces and voices, and decreased levels of interest (Elgar et al., 2004, Lundy et al., 1996). Furthermore, it is also possible that biological symptoms of depression including sleep disturbance, low energy and loss of interest may each contribute to decreased involvement in interactions with the infant.

## 5 Conclusion

This study shows that early playful paternal behaviour with young infants differs between fathers with and without diagnosed depression. These behaviours specifically reduced excitatory playfulness, gentle tactile stimulation, and less active engagement could be potential targets for future family interventions, early in life. Furthermore, this new evidence of associations between depression and fathers play behaviours with their young infants highlights the need for the continued development and application of father-focussed measures. Studying fathers who are experiencing difficulties in their relationship with their infants is an important undertaking.

## Footnotes

* Note: Physical play and playful excitation are coded separately. However, they are introduced together as they may occur simultaneously, precede, or follow one another, within the same interactive context.

## References

[bib1] Arco C.M. (1983). Pacing of playful stimulation to young infants: similarities and differences in maternal and paternal communication. Infant Behav. Dev..

[bib2] Barker B., Iles J.E., Ramchandani P.G. (2017). Fathers, fathering and child psychopathology. Curr. Opin. Psychol..

[bib3] Basco M.R., Bostic J.Q., Davies D., Rush J., Witte B., Hendrickse W. (2000). Methods to improve diagnostic accuracy in a community mental health setting. Am. J. Psychiatry.

[bib4] Belsky J. (1979). Mother-father-infant interaction: a naturalistic observational study. Dev. Psychol..

[bib5] Belsky J., Gilstrap B., Rovine M. (1984). The Pennsylvania infant and family development project, I: stability and change in mother-infant and father-infant interaction in a family setting at one, three, and nine months. Child Dev..

[bib6] Bridges L.J., Connell J.P. (1991). Consistency and inconsistency in infant emotional and social interactive behavior across contexts and caregivers. Infant Behav. Dev..

[bib7] Bridges L.J., Grolnick W.S., Connell J.P. (1997). Infant emotion regulation with mothers and fathers. Infant Behav. Dev..

[bib8] Bronstein P. (1984). Differences in mothers' and fathers' behaviors toward children: a cross-cultural comparison. Dev. Psychol..

[bib9] Cabrera N.J., Fitzgerald H.E., Bradley R.H., Roggman L. (2014). The ecology of Father-child relationships: an expanded model. J. Fam. Theory Rev..

[bib10] Cameron E.E., Sedov I.D., Tomfohr-Madsen L.M. (2016). Prevalence of paternal depression in pregnancy and the postpartum: an updated meta-analysis. J. Affect. Disord..

[bib11] Carson J., Burks V., Park R.D. (1993).

[bib12] Clarke-Stewart K.A. (1978). And daddy makes three: the father's impact on mother and young child. Child Dev..

[bib13] Cohn J.F., Matias R., Tronick E.Z., Connell D., Lyons-Ruth K. (1986). Face-to-face interactions of depressed mothers and their infants. New Dir. Child Adolesc. Dev..

[bib14] Cox J.L., Holden J.M., Sagovsky R. (1987). Detection of postnatal depression: development of the 10-item Edinburgh postnatal depression scale. Br. J. Psychiatry.

[bib15] Craig L. (2006). Does father care mean fathers share? A comparison of how mothers and fathers in intact families spend time with children. Gend. Soc..

[bib16] Crawley S.B., Sherrod K.B. (1984). Parent-infant play during the first year of life. Infant Behav. Dev..

[bib17] Diener M.L., Mangelsdorf S.C., McHale J.L., Frosch C.A. (2002). Infants' behavioral strategies for emotion regulation with fathers and mothers: associations with emotional expressions and attachment quality. Infancy.

[bib18] Dixon S.D., Yogman M., Tronick E., Adamson L., Als H., Brazelton T.B. (1981). Early infant social interaction with parents and strangers. J. Am. Acad. Child Psychiatry.

[bib19] Edmondson O.J.H., Psychogiou L., Vlachos H., Netsi E., Ramchandani P.G. (2010). depression in fathers in the postnatal period: assessment of the Edinburgh Postnatal depression scale as a screening measure. J. Affect. Disord..

[bib20] Edward K.-l., Castle D., Mills C., Davis L., Casey J. (2014). An integrative review of paternal depression. Am. J. Men's Health.

[bib21] Elgar F.J., McGrath P.J., Waschbusch D.A., Stewart S.H., Curtis L.J. (2004). Mutual influences on maternal depression and child adjustment problems. Clin. Psychol. Rev..

[bib22] Feldman R. (2003). Infant-mother and infant-father synchrony: the coregulation of positive arousal. Infant Ment. Health J..

[bib23] Feldman R., Eidelman A.I. (2003). Skin-to-skin contact (Kangaroo care) accelerates autonomic and neurobehavioural maturation in preterm infants. Dev. Med. Child Neurol..

[bib24] Ferber S.G. (2004). The nature of touch in mothers experiencing maternity blues: the contribution of parity. Early Hum. Dev..

[bib25] Ferber S.G., Feldman R., Makhoul I.R. (2008). The development of maternal touch across the first year of life. Early Hum. Dev..

[bib26] Field T.M., Healy B., Goldstein S., Guthertz M. (1990). Behaviour-state matching and synchrony in mother-infant interactions of non-depressed versus depressed dyads. Dev. Psychol..

[bib27] Field T.M., Hossain Z., Malphurs J. (1999). “Depressed” fathers' interactions with their infants. Infant Ment. Health J..

[bib28] First M.B., Gibbon M., Spitzer R.L., Williams J.B. (2002). Structured Clinical Interview for DSM-IV-TR Axis I Disorders, Research Version, Non-patient Edition. (SCID-I/NP).

[bib29] Hallers-Haalboom E.T., Groeneveld M.G., van Berkel S.R., Endendijk J.J., van der Pol L.D., Linting M. (2017). Mothers' and fathers' sensitivity with their two children: a longitudinal study from infancy to early childhood. Dev. Psychol..

[bib30] Hanington L., Ramchandani P., Stein A. (2010). Parental depression and child temperament: assessing child to parent effects in a longitudinal population study. Infant Behav. Dev..

[bib31] Herrera E., Reissland N., Shepherd J. (2004). Maternal touch and maternal child-directed speech: effects of depressed mood in the postnatal period. J. Affect. Disord..

[bib32] Hertenstein M.J., Campos J.J. (2001). Emotion regulation via maternal touch. Infancy.

[bib33] Kane P., Garber J. (2009). Parental depression and child externalizing and internalizing symptoms: unique effects of father's symptoms and perceived conflict as a mediator. J. Child Fam. Stud..

[bib34] Koester L.S., Papousek H., Papousek M. (1989). Patterns of rhythmic stimulation by mothers with three-month-olds: a cross-modal comparison. Int. J. Behav. Dev..

[bib35] Labrell F. (1994). A typical interaction behaviour between fathers and toddlers: teasing. Early Dev. Parent..

[bib36] Lamb M.E. (1977). Father-infant and mother-infant interaction in the first year of life. Child Dev..

[bib37] Lamb M.E., Frodi A.M., Hwang C.P., Frodi M., Steinberg J. (1982). Mother-and father-infant interaction involving play and holding in traditional and nontraditional Swedish families. Dev. Psychol..

[bib38] Lewis S.N., West A.F., Stein A., Malmberg L.E.L.E., Bethell K.L.E., Barnes J.L.E. (2009). A comparison of father-infant interaction between primary and non-primary care giving fathers. Child Care Health Dev..

[bib39] Lundy B.L. (2002). Paternal socio-psychological factors and infant attachment: the mediating role of synchrony in father-infant interactions. Infant Behav. Dev..

[bib40] Lundy B.L., Field T., Pickens J. (1996). Newborns of mothers with depressive symptoms are less expressive. Infant Behav. Dev..

[bib41] MacDonald K., Park R.D. (1986). Parent-child physical play: the effects of sex and age of children and parents. Sex Roles.

[bib42] Malphurs J.E., Pickens J., Pelaez-Nogueras M., Yando R., Bendell D. (1996). Altering withdrawn and intrusive interaction behaviors of depressed mothers. Infant Ment. Health J..

[bib43] Malphurs J.E., Raag T., Field T., Picken J., Palaez-Nogueras M. (1996). Touch by intrusive and withdrawn mothers with depressive symptoms. Early Dev. Parent..

[bib44] McBride B.A., Schoppe S.J., Rane T.R. (2002). Child characteristics, parenting stress, and parental involvement: fathers versus mothers. J. Marriage Fam..

[bib45] McElwain N.L., Volling B.L. (1999). Depressed mood and marital conflict: relations to maternal and paternal intrusiveness with one-year-old infants. J. Appl. Dev. Psychol..

[bib46] Moreno A.J., Posada G.E., Goldyn D.T. (2006). Presence and quality of touch influence coregulation in mother-infant dyads. Infancy.

[bib47] Murray L., Fiori-Cowley A., Hooper R., Cooper P. (1996). The impact of postnatal depression and associated adversity on early mother-infant interactions and later infant outcome. Child Dev..

[bib48] Nath S., Russell G., Kuyken W., Psychogiou L., Ford T. (2016). Does father–child conflict mediate the association between fathers' postnatal depressive symptoms and children's adjustment problems at 7 years old?. Psychol. Med..

[bib49] Paquette D., Dumont C. (2013). Is father–child rough-and-tumble play associated with attachment or activation relationships?. Early Child Dev. Care.

[bib50] Parfitt Y., Pike A., Ayers S. (2013). The impact of parents' mental health on parent-baby interaction: a prospective study. Infant Behav. Dev..

[bib51] Paulson J.F., Bazemore S.D. (2010). Prenatal and postpartum depression in fathers and its association with maternal depression. [Review]. J. Am. Med. Assoc..

[bib52] Paulson J.F., Dauber S., Leiferman J.A. (2006). Individual and combined effects of postpartum depression in mothers and fathers on parenting behavior. Pediatrics.

[bib53] Pelaez-Nogueras M., Gewirtz J.L., Field T., Cigales M., Malphurs J., Clasky S. (1996). Infants' preference for touch stimulation in face-to-face interactions. J. Appl. Dev. Psychol..

[bib54] Pellegrini A.D., Smith P.K. (1998). Physical activity play: the nature and function of a neglected aspect of playing. Child Dev..

[bib55] Polan H.J., Ward M.J. (1994). Role of the mother's touch in failure to thrive: a preliminary investigation. J. Am. Acad. Child Adolesc. Psychiatry.

[bib56] Power T.G. (1985). Mother-and father-infant play: a developmental analysis. Child Dev..

[bib57] Ramchandani P.G., Psychogiou L., Vlachos H., Iles J., Sethna V., Netsi E. (2011). Paternal depression: an examination of its links with father, child and family functioning in the postnatal period. Depress. Anxiety.

[bib58] Roggman L., Boyce L.K., Cook G.A., Christiansen K., Jones D. (2004). Playing with daddy: social toy play, early head start and developmental outcomes. Fathering.

[bib59] Rosenbaum P.R., Rubin D.B. (1985). Constructing a control group using multivariate matched sampling methods that incorporate the propensity score. Am. Stat..

[bib60] Rubin D.B. (1979). Using multivariate matched sampling and regression adjustment to control bias in observational studies. J. Am. Stat. Assoc..

[bib61] Segrin C. (2000). Social skills deficits associated with depression. Clin. Psychol. Rev..

[bib62] Sethna V., Murray L., Netsi E., Psychogiou L., Ramchandani P.G. (2015). Paternal depression in the postnatal period and early father–infant interactions. Parent. Sci. Pract..

[bib63] Sethna V., Murray L., Ramchandani P. (2012). Depressed fathers' speech to their 3-month-old infants: a study of cognitive and mentalizing features in paternal speech. Psychol. Med..

[bib64] Shields M.J., Sparling J. (1993). Fathers' play and touch behavior with their three-month-old infants. Phys. Occup. Ther. Pediatr..

[bib65] Stack D.M., Muir D.W. (1992). Adult tactile stimulation during face-to-face interactions modulates five-month-olds' affect and attention. Child Dev..

[bib66] Stevenson M.B., Leavitt L.A., Thompson R.H., Roach M.A. (1988). A social relations model analysis of parent and child play. Dev. Psychol..

[bib67] Stgeorge J., Freeman E. (2017). Measurement of father-child rough and tumble play and its relations to child behavior. Infant Ment. Health J..

[bib68] Tamis-LeMonda C.S. (2004). Conceptualizing fathers' roles: playmates and more. Hum. Dev..

[bib69] Tamis-LeMonda C.S., Shannon J.D., Cabrera N.J., Lamb M.E. (2004). Fathers and mothers at play with their 2- and 3-year-olds: contributions to language and cognitive development. Child Dev..

[bib70] Teti D.M., Bond L.A., Gibbs E.D. (1988). Mothers, fathers, and siblings: a comparison of play styles and their influence upon infant cognitive level. Int. J. Behav. Dev..

[bib71] Tronick E.Z., Field T. (1985). Touch in Mother-infant Interaction. Touch in Early Development.

[bib72] Tronick E.Z., Cohn J.F. (1989). Infant-mother face-to-face interaction: age and gender differences in coordination and the occurrence of miscoordination. Child Dev..

[bib73] Vismara L., Rollè L., Agostini F., Sechi C., Fenaroli V., Molgora S. (2016). Perinatal parenting stress, anxiety, and depression outcomes in first-time mothers and fathers: a 3- to 6-months postpartum follow-up study. Front. Psychol..

[bib74] Weinberg M.K., Tronick E.Z. (1998). The impact of maternal psychiatric illness on infant development. J. Clin. Psychiatry.

[bib75] Weinraub M., Frankel J. (1977). Sex differences in parent-infant interaction during free play, departure, and separation. Child Dev..

[bib76] Weiss S.J. (1988). Touch. Annu. Rev. Nurs. Res..

[bib77] Weiss S.J., Wilson P. (2006). Origins of tactile vulnerability in high-risk infants. Adv. Neonatal Care.

[bib78] Yarrow L.J., MacTurk R.H., Vietze P.M., McCarthy M.E., Klein R.P., McQusiton S. (1984). Developmental course of parental stimulation and its relationship to mastery motivation during infancy. Dev. Psychol..

[bib79] Yogman, M., 1977. The goals and structure of face-to-face interaction between infants and fathers. Paper presented at the Society for Research in Child Development.

[bib80] Yogman M. (1981). Games fathers and mothers play with their infants. Infant Ment. Health J..

[bib81] Zanarini M.C., Skodol A.E., Bender D., Dolan R., Sanislow C., Schaefer E. (2000). The collaborative longitudinal personality disorders study: reliability of axis I and II diagnoses. J. Pers. Disord..

[bib82] Zaslow M.J., Pedersen F.A., Cain R.L., Suwalsky J.T., Kramer E.L. (1985). Depressed mood in new fathers: associations with parent-infant interaction. Genet. Soc. Gen. Psychol. Monogr..

